# Physical frailty, depression and multimorbidity among older adults in Lima, Peru: a cross-sectional study

**DOI:** 10.1186/s12877-026-07850-8

**Published:** 2026-06-20

**Authors:** Oscar Flores-Flores, Alejandro Zevallos-Morales, Suzanne L. Pollard, Trishul Siddharthan, Alden L. Gross, José F. Parodi, Joseph J. Gallo, John R. Hurst, William Checkley

**Affiliations:** 1 Centro de Investigación del Envejecimiento (CIEN), Facultad de Medicina Humana, Universidad de San Martin de Porres, Alameda del Corregidor 1531, la Molina, Lima, 15024 Peru; 2 Department of International Health, Johns Hopkins University Bloomberg School of Public Health, Baltimore, MD USA; 3 Asociacion Benefica PRISMA, Lima, Peru; 4 Center for Global Non-Communicable Disease Research and Training, School of Medicine, Johns Hopkins University, Baltimore, MD USA; 5 Department of Medicine, Division of Pulmonary and Critical Care, School of Medicine, Johns Hopkins University, Baltimore, MD USA; 6 Division of Pulmonary and Critical Care, Miller School of Medicine, University of Miami, Miami, FL USA; 7 Department of Epidemiology, Johns Hopkins Bloomberg School of Public Health, Baltimore, MD USA; 8 Center on Aging and Health, Johns Hopkins University, Baltimore, MD USA; 9 Department of Mental Health, Johns Hopkins Bloomberg School of Public Health, Baltimore, MD USA; 10 UCL Respiratory, University College London, London, UK

**Keywords:** Multimorbidity, Depression, Physical Frailty, Older Adults

## Abstract

**Background:**

Multimorbidity, frailty, and depression frequently co-occur in older adults and contribute to adverse health outcomes. This study identified classes of physical multimorbidity and examined their association with depression, and whether this association differs by frailty status.

**Methods:**

We conducted a cross-sectional study in low-resource urban areas of Lima, Peru. We included adults aged ≥ 60 years, and assessed the presence of physical multimorbidity (COPD, asthma, chronic bronchitis, heart disease, diabetes, arthritis, and hypertension), physical frailty phenotype, and depression (Patient Health Questionnaire-9 score ≥ 7). We applied latent class analysis to categorize participants into physical multimorbidity classes. Subsequently, we conducted Poisson regression with robust variance to assess the relationship between somatic multimorbidity classes and depression. We studied the role of frailty by stratifying the analysis according to the presence or absence of frailty.

**Results:**

Among 1029 participants (mean age 68.9 years; 44.6% women), 201 (19.5%) had depression. Three multimorbidity classes were identified: cardiometabolic, multisystem, and apparently healthy. Only the multisystem class was associated with a higher prevalence of depressive symptoms compared to the apparently healthy class, after adjusting for sex, age, education, and socio-economic position (adjusted Prevalence Ratio, aPR = 1.80, 95% CI: 1.02–3.06). Among individuals with frailty, depression was more prevalent in the multisystem class (aPR 6.25, 95% CI 1.28–30.63), whereas no significant association was observed among non-frail participants (aPR 1.58, 95% CI 0.83–3.00).

**Conclusions:**

The association between multisystem multimorbidity and depressive symptoms was stronger among individuals with frailty. Considering frailty alongside multimorbidity may improve identification of subgroups with greater depressive burden, particularly in resource-constrained settings.

**Supplementary Information:**

The online version contains supplementary material available at https://doi.org/10.1186/s12877-026-07850-8.

## Introduction

Multimorbidity, defined as the coexistence of two or more chronic diseases, is particularly prevalent among older adults [1]. In this study, we focused specifically on physical (somatic) chronic conditions while mental health conditions such as depression are considered as outcomes. Managing multiple health conditions presents significant challenges, affecting the well-being of both individuals and caregivers [2–4]. Traditional medical training, which often focuses on single diseases and treatment, is ill-equipped to address the complex interactions between multiple chronic conditions [3, 5]. As people age, multimorbidity becomes more common. The World Health Organization report on Ageing and Health emphasizes that the consequences of multimorbidity extend beyond the additive effects of individual diseases, underscoring the need for a more comprehensive understanding of its broader impact [6–8].

Late life depression is one of the most common mental disorders [9]. Clinically significant depressive symptoms affect 15% of community-dwelling older adults [10]. In a systematic review, depression was two to three times more likely in people with physical multimorbidity [11]. Depression itself is an independent predictor of various adverse outcomes, including disability and mortality [12]. However, fewer studies have examined how depression varies across specific patterns of physical multimorbidity, and how heterogeneity within multimorbid populations may shape this association [13, 14].

Physical frailty is a syndrome characterized by reduced strength, endurance, and physiological function, leading to increased vulnerability to stressors [15]. It affects approximately 10% of older adults living in the community [16] and it is associated with adverse health outcomes such as falls, disability, hospitalization, and death [17].

At the population level, physical multimorbidity, frailty, and depression represent a set of interrelated but distinct conditions. While much of the literature has focused on pairwise associations among multimorbidity, frailty, and depression [18] a growing body of work has begun to examine the role of frailty in shaping mental health outcomes among older adults with multiple chronic conditions [19–22]. However, less is known about whether frailty helps identify subgroups of older adults with physical multimorbidity who experience a higher burden of depressive symptoms, particularly in low-resource settings.

Based on a large cross-sectional study among older adults conducted in low-resource urban settings in Lima, Peru, our objectives were to identify patterns of physical multimorbidity and their association with depression, and evaluate whether frailty modifies the relationship between physical multimorbidity patterns and depression.

## Methods

### Study design and participants

The Global Excellence in COPD Outcomes (GECo) study [23, 24] was cross-sectional, community-based study that collected data from adults aged ≥ 40 years in an age- and sex-stratified sample from three sites: Bhaktapur, Nepal; Lima, Peru; and Nakaseke, Uganda, between 2018 and 2020. The GECo sample comprised individuals aged ≥ 40 years, capable of undergoing spirometry tests, and living full-time within the designated areas. GECo excluded anyone who reported being pregnant, had active pulmonary tuberculosis or was undergoing treatment for tuberculosis, or had conditions that made spirometry unsafe, such as recent eye, chest, or abdominal surgery, myocardial infarction within the previous three months, or a blood pressure higher than 180/100 mm Hg at the time of evaluation. Although spirometry was performed as part of the study protocol, COPD was not an inclusion criterion, and a prior diagnosis of COPD did not disqualify participants.

For the present study, we selected a sub-sample of adults, aged ≥ 60 years from an urban low-income setting in Lima, Peru. This decision was made because frailty assessments were only conducted in the Peru site.

### Operational definitions

Physical (somatic) multimorbidity was defined as the presence of two or more of eight chronic conditions: COPD, asthma, chronic bronchitis, heart disease, diabetes, arthritis, hypertension, and obesity. The definition and operational details for each condition are provided in Table 1.

**Table 1:** Physical chronic conditions and operationalization

	Condition	Operational definition
1	COPD	Ratio of postbronchodilator forced expiratory volume in 1 s (FEV_1_) to forced vital capacity (FVC) less than the fifth percentile of the Global Lung Function Initiative mixed ethnic population reference lower limit of normal (LLN).
2	Asthma	Two or more: (1) having a diagnosis of asthma by a physician, (2) self-reported wheezing in the last 12 months, (3) self-reported asthma medication usage in the last 12 months.
3	Chronic Bronchitis	Cough with phlegm most days of the week and nearly daily for three consecutive months during the last 2 years.
4	Heart Disease	Self-report physician diagnosis of heart failure, arrhythmia, or coronary artery disease (i.e., angina and myocardial infarction).
5	Diabetes	Self-report physician diagnosis of diabetes.
6	Arthritis	Self-report health provider diagnosis of arthritis.
7	Hypertension	Self-report health provider diagnosis of hypertension OR high blood pressure assessment (140/90 mmHg) during study visit.
8	Obesity	Body Mass Index (BMI) > = 30 kg/m^2^

Physical Frailty Phenotype [15] was operationalized using a modified version of the Fried phenotype. Weight loss was defined as self-reported unintentional loss of ≥ 5 kg in the past 12 months. Weakness by hand grip strength in the lowest sex- and body-mass-index (BMI)-adjusted quintile. Exhaustion was determined through self-reported responses to three items from the Center for Epidemiological Studies Depression (CES–D) scale [25]. Slowness was assessed by 4-meter gait speed, with values in the lowest quintile adjusted for sex and standing height [26]. Low physical activity was defined by scores in the lowest quintile score of the Spanish-adapted Physical Activity Scale for the Elderly (PASE) [27, 28]. Given that the exhaustion component was based on items from a depression scale, and to reduce potential overlap with the outcome of depressive symptoms, we conducted analyses excluding this component [29]. In these analyses, the frailty score ranged from 0 to 4, and participants were classified as frail if they met three or more of the remaining criteria. As a sensitivity analysis, we also examined frailty using the full five-criterion Fried phenotype, with individuals meeting three to five criteria classified as frail; these results are presented in the Additional File, Table S2.

Depression was assessed using the Patient Health Questionnaire-9 (PHQ-9), a validated nine-item Likert self-report scale with scores ranging from 0 to 27 [30]. Each item has four response options: 0 = not at all, 1 = several days, 2 = more than half the days, and 3 = nearly every day. The PHQ-9 has been validated in the Peruvian adult population [31, 32]. For this study, we selected a PHQ-9 ≥ 7 to define clinically significant depression, based on previous research in Peru indicating this threshold offers optimal sensitivity and specificity [33].

During the initial phase of data collection, we used sequential testing [34, 35], in which participants who scored ≥ 2 points on the PHQ-2 were administered the remaining seven items of the PHQ-9. In our sample, this approach was used in only 3.5% of cases. We also compared mean PHQ-9 scores between participants who underwent sequential testing and those who completed the full PHQ-9 (see, Additional File 1, Table S1).

Covariates included in our models were sex, age, and educational attainment (categorized as no education, incomplete primary education, completed primary education, any secondary education, and any higher education), and socioeconomic status (SES), based on the material composition of household’s flooring and roofing.

### Study procedures

Trained fieldworkers conducted in-person demographic surveys in Spanish to collect information on socioeconomic status and medical history in participant’s homes. Data were recorded using REDCap software on tablets. Blood pressure was obtained at the beginning of the visit. Weight and height were each recorded three times. Hand grip strength was assessed three times using the dominant hand with a JAMAR hydraulic dynamometer (Akron, OH, USA), 4-meter gait speed was evaluated twice. Spirometry tests were carried out using the Easy-On-Air spirometer (ndd, Zurich, Switzerland) following standard recommendations [36], both before and after administering 400 mcg of inhaled salbutamol [37, 38].

### Statistical methods

We first examined means and proportions of the general characteristics of the study sample. Next, we used latent class analysis (LCA) to identify classes or patterns of physical multimorbidity. LCA is a statistical method that identifies unobserved (latent) classes within a population based on individual responses to categorical indicator variables [39, 40]. It is a person-centered, model-based clustering technique that classifies individuals into mutually exclusive groups sharing similar patterns of observed characteristics [39].

For this analysis, we used data from 1,029 individuals to evaluate the optimal number of latent classes based on eight chronic conditions: COPD, asthma, chronic bronchitis, heart disease, diabetes, arthritis, hypertension, and obesity. We estimated models with 2, 3, and 4 latent classes and compared them sequentially using several goodness-of-fit statistics, including the Akaike Information Criterion (AIC) and Bayesian Information Criterion (BIC) [41, 42], We also employed the Lo-Mendel-Rubin (LMR) likelihood ratio test and the Bootstrapped Likelihood ratio test (BLRT), both of which test the hypothesis that a model with k classes does not provides a better fit than a model with k − 1 classes. LCA analyses were conducted using MPLUS version 8.1.

After determining the optimal number of latent classes had been identified, each participant was assigned to the class for which they had the highest posterior probability, i.e., their most likely class membership. We then used Poisson regression models with robust variance estimators to estimate prevalence ratios (PRs) and 95% confidence intervals (CIs) for the association between class membership and depression using Stata version 17 (StataCorp LLC, College Station, TX, USA). We conducted crude and adjusted models. The final model was adjusted for sex, age, educational level, and SES. To evaluate effect modification by frailty status, we conducted a likelihood ratio test comparing models with and without the interaction term.

## Results

### Sample characteristics

**Fig. 1:**
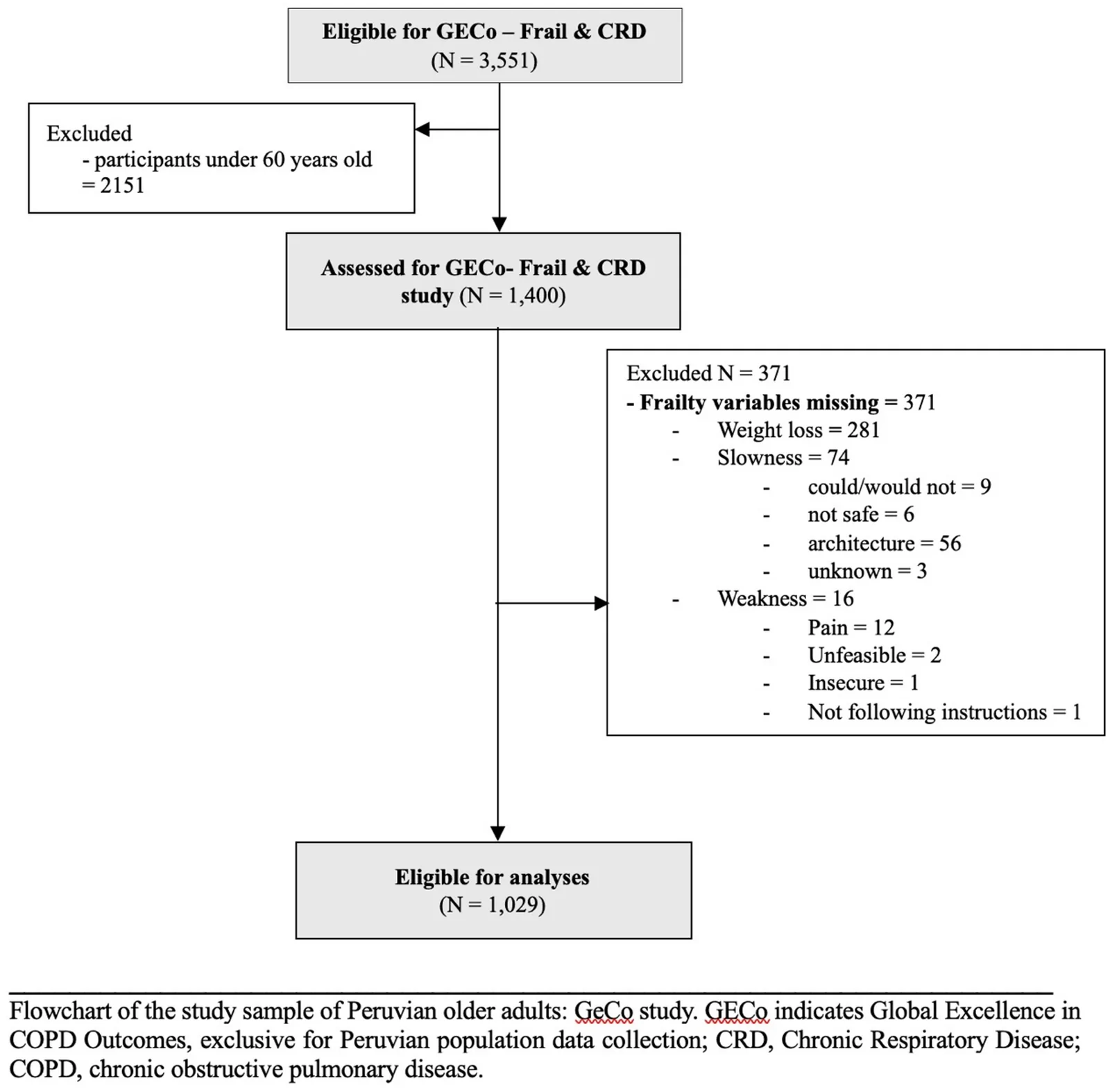
Flowchart of the participants

Figure 1 shows the flow of study participants. In Table 2, we summarize the general characteristics of the 1,029 participants. Mean (SD) age of participants was 68.9 (6.3) years, and 55.4% were men. Educational attainment was low, with only 8.4% reporting any higher education.

**Table 2:** General characteristic of the 1029 study participants

Characteristics		
Age, mean (SD) (years)		68.9 (6.3)
Age, range (years)		60–95
Age categories, *n* (%)	60.0–69.9 years	592 (57.5%)
	70.0–79.9 years	377 (36.6%)
	80 or more years	60 (5.8%)
Sex, *n* (%)		
	Female	459 (44.6%)
BMI, mean (SD) kg/m^2^		29.4 (4.6)
Education Level, *n* (%)	None	70 (6.8%)
	Incomplete primary school	275 (26.8%)
	Complete primary school	228 (22.2%)
	Any secondary school	369 (35.9%)
	Any higher education	86 (8.4%)
Socioeconomic position, *n* (%)	Lowest	119 (11.6%)
	Medium	374 (36.3%)
	Highest	536 (52.1%)
COPD, *n* (%)		
	Yes	32 (3.1%)
Asthma, *n* (%)		
	Yes	45 (4.4%)
Chronic bronchitis, *n* (%)		
	Yes	108 (10.5%)
Heart Disease, *n* (%)		
	Yes	56 (5.4%)
Diabetes, *n* (%)		
	Yes	165 (16.0%)
Arthritis, *n* (%)		
	Yes	77 (7.5%)
Hypertension, *n* (%)		
	Yes	543 (52.8%)
Obesity, *n* (%)		
	Yes	411 (39.9%)
Depressive symptoms, *n* (%)	0–6 None or minimal	828 (80.5%)
	7 or more, mod-severe	201 (19.5%)
PHQ score total, mean (SD)		3.6 (3.8)
Frailty criteria		
*Criteria 1 - weight loss*,* n (%)*		
	Yes	178 (17.3%)
*Criteria 2 – exhaustion*,* n (%)*		
	Yes	162 (15.7%)
*Criteria 3 – slowness*,* n (%)*		
	Yes	214 (20.8%)
*Criteria 4 - low physical activity*,* n (%)*		
	Yes	205 (19.9%)
*Criteria 5 – weakness*,* n (%)*		
	Yes	235 (22.8%)
Presence Physical Frailty [5 criteria]		
	Yes	86 (8.4%)
Presence Physical Frailty [4 criteria] *		
	Yes	56 (5.4%)
Physical Frailty categories [5 criteria]	Robust	401 (39.0%)
	Pre-frail	542 (52.7%)
	Frail	86 (8.4%)
Physical Frailty categories [4 criteria]	Robust	452 (43.9%)
	Pre-frail	521 (50.6%)
	Frail	56 (5.4%)
Physical multimorbidity**		
	Yes	424 (41.2%)
Number of physical conditions	0	208 (20.2%)
	1	397 (38.6%)
	2	277 (26.9%)
	3	110 (10.7%)
	4	30 (2.9%)
	5	7 (0.7%)

* Excluding exhaustion criteria of Physical Frailty Phenotype

** Physical multimorbidity includes having 2 or more of the following conditions: COPD, asthma, chronic bronchitis, heart disease, diabetes, arthritis, hypertension, and obesity

Physical multimorbidity was identified in over two-fifths of the sample 41.2% (95%CI 38.2–44.2). The most prevalent conditions were hypertension (52.8%), obesity (39.9%), and diabetes (16.0%). Mean (SD) PHQ-9 score was 3.6 (3.8) and 201 participants (19.5%) had clinically significant depression.

Using a four-criterion frailty definition that excluded exhaustion, physical frailty was present in 5.4% of participants. When exhaustion was included, applying the standard five-criterion definition, frailty prevalence increased to 8.4%. Among individual frailty components, weakness was the most commonly observed (22.8%).

### Identifying multimorbidity patterns using latent class analysis

In Table 3, based on model fit statistics (AIC, BIC, LMR, and BLRT) and model interpretability, we selected a three-class model as optimal.

**Table 3:** Fit Indices for the Latent Class Analyses of the physical multimorbidity models among 1029 participants

Classes	#parameters	AIC	BIC	LMR(*p*)	BLRT(*p*)
2	17	6028.705	6112.623	0.0158	0.000
3	26	6001.462	6129.807	0.0277	0.000
4	35	5997.230	6170.002	0.2855	0.0612

*AIC* Akaike information criterion, *BIC*  Bayesian information criterion, *LMR* (*p*)  *P*-value of Lo-Mendel Rubin tests, *BLRT* (*p*)  *P*-value of parametric bootstrapped likelihood ratio test

In Table 4, we present the estimated conditional probabilities for each health condition, as well as class membership probabilities both from the model and after assigning individuals by their highest posterior probability. Differences between model-estimated and assigned probabilities were less than 10%, indicating good classification accuracy.

**Table 4:** Estimated conditional probabilities, latent class probabilities, and proportion of frailty and depression among the 3-class model (*n* = 1029)

Variables	Prevalence		Class 1	Class 2	Class 3
		‘cardiometabolic’		‘multisystem’	‘Apparently healthy’
COPD	3.1%	0.00 [0.00]		0.23 [0.08]	0.05 [0.03]
Asthma	4.4%	0.00 [0.00]		0.54 [0.17]	0.04 [0.03]
C. bronchitis	10.5%	0.07 [0.02]		0.40 [0.10]	0.12 [0.03]
Heart Disease	5.4%	0.08 [0.03]		0.20 [0.08]	0.00 [0.00]
Diabetes	16.0%	0.20 [0.03]		0.29 [0.09]	0.08 [0.05]
Arthritis	7.5%	0.07 [0.02]		0.21 [0.09]	0.06 [0.03]
Hypertension	52.8%	0.71 [0.13]		0.75 [0.12]	0.20 [0.12]
Obesity	39.9%	0.49 [0.05]		0.46 [0.10]	0.24 [0.10]
Latent class probabilities (model)	-	0.58		0.06	0.36
Latent class (most likely membership)	-	0.67		0.04	0.28
*Frailty-4 criteria, % (model)	5.4%	6.6%		5.6%	3.6%
**Frailty-4 criteria,% (after membership assignment)	5.4%	5.9%		5.3%	4.4%
***Depression, % (model)	19.5%	23.4%		31.2%	11.6%
****Depression, % (after membership assignment)	19.5%	21.1%		29.0%	14.8%

*No statistical difference between groups (Chi-square), overall test and by pairs (*p* > 0.05)

** No statistical difference between groups (Chi-square), overall test and by pairs (*p* > 0.05)

***Overall test Chi-square (*p* < 0.05). Class1vs2 (*p* = 0.38), Class1vs3 (*p* < 0.05), Class2vs3 (*p* < 0.05)

**** Overall test Chi-square (*p* < 0.05). Class1vs2 (*p* > 0.05), Class1vs3 (*p* < 0.05), Class2vs3 (*p* < 0.05)

We identified three distinct multimorbidity classes. The cardiometabolic class was the most common, with a model-estimated membership probability of 58%, rising to 67% after posterior probability assignment. This class is characterized by a high probability of diabetes, obesity, and hypertension, with low probabilities for the other conditions. The multisystem physical multimorbidity class represented 6% of the sample in the model, decreasing to 4% after assignment. We label as multisystem physical multimorbidity for the disease involvement across multiple organ systems. This class included participants with high probabilities across nearly all assessed conditions, including diabetes, hypertension, and obesity, alongside respiratory conditions, heart disease, and arthritis, indicating a high overall burden of disease across multiple organ systems. Finally, the apparently healthy class had the lowest prevalence for all reported conditions studied. The model estimated 36% of participants in this class, which declined to 28% after assignment.

In Table 4, we summarize the prevalence of physical frailty across classes: 6.6% in the cardiometabolic class, 5.6% in the multisystem class, and 3.6% in the apparently healthy class. After assignment, these values were 5.9%, 5.3%, and 4.4%, respectively. In contrast, depression showed significant variation across classes: depression was present in 23.4% of the cardiometabolic class, 31.2% of the multisystem class, and 11.6% of the apparently healthy class. After assignment, the corresponding values were 21.1%, 29.0%, and 14.8%, respectively.

### Role of physical frailty in multimorbidity and depression

In Table 5, we summarize the association between multimorbidity classes and depression. The association between multimorbidity class and depression was modified by frailty status. Among frail participants, those in the multisystem class had a 6.25 times (95%CI 1.28–30.63) higher prevalence of depression compared to those in the apparently healthy class. Among non-frail participants, there was no significant difference in depression prevalence between the multisystem and apparently healthy classes (PR = 1.58; 95% CI: 0.83–3.00). A sensitivity analysis using the full five-criterion definition of frailty (including exhaustion) yielded similar patterns (Additional File, Table S2).

**Table 5:** Prevalence ratio (PR) associations between physical multimorbidity classes and depression

Class membership	Depression (vs. No depression)	Depression (vs. No depression)
	Unadjusted model	Adjusted model*
	PR (95%CI)	PR (95%CI)
Ap. healthy	reference	reference
multisystem	**1.96 (1.11–3.46)**	**1.80 (1.02–3.16)**
cardiometabolic	**1.43 (1.05–1.94)**	1.35 (0.99–1.84)
Among those with frailty		
Ap. healthy	reference	reference
multisystem	**6.5 (1.79–25.53)**	**6.25 (1.28–30.63)**
cardiometabolic	1.90 (0.48–7.51)	2.16 (0.60–7.72)
Among those without frailty		
Ap. healthy	reference	reference
multisystem	1.69 (0.90–3.19)	1.58 (0.83-3.00)
cardiometabolic	**1.39 (1.02–1.92)**	1.32 (0.96–1.81)

Frailty excluding exhaustion criteria

*PR* Prevalence Ratio

Values in bold indicate statistical significance at *p* < 0.05

*Adjusted by sex, age, educational level, and socio-economic position

## Discussion

Using a latent class model, we identified three classes of physical multimorbidity in among older adults in a resource-poor setting in Lima, Peru: a cardiometabolic class, a multisystem class and an apparently healthy class. Nearly 60% of participants were in the cardiometabolic class. Those in the multisystem class showed a higher prevalence of depression compared to the apparently healthy class. Furthermore, frailty amplified the probability of depression among participants in the multisystem class.

Our findings indicate a 41% prevalence of physical multimorbidity in this population, consistent with estimates from other studies from low to middle-income countries [1, 43]. While the naming of classes varies across studies, the patterns are broadly comparable. The cardiometabolic class, defined by high prevalence of diabetes, hypertension, and obesity, reflects patterns seen globally [44], and is strongly linked to adverse health outcomes. The multisystem class represents individuals with high probabilities across a wide range of chronic conditions, suggesting a particularly vulnerable subgroup with extensive multimorbidity [45].

The higher prevalence of depression in the multisystem class may not be attributable solely to the number of chronic conditions, but also to disease severity, functional decline, or other unmeasured factors. The association between multimorbidity and depression is increasingly understood as bidirectional and shaped by a complex interplay of biological, psychosocial, and care-related factors that contribute to accelerated aging [46, 47]. Symptoms such as pain, mobility issues, and poor sleep have been proposed as potential linking multimorbidity with depression [48]. Taken together, the mechanisms linking multimorbidity and depression are widely recognized as operating across multiple levels, including aging-related biological processes such as chronic inflammation and neuroendocrine and autonomic dysregulation (e.g., autonomic nervous system imbalance and hypothalamic–pituitary–adrenal axis overactivity) [46], as well as broader determinants of health, including lifestyle behaviors, chronic disease management, and access to and continuity of care. Although these variables were not directly measured in the present study, frailty serves as an integrative marker of vulnerability that helps identify individuals with physical multimorbidity who are at higher risk of adverse mental health outcomes.

Some studies [20, 49] examined frailty alongside depression and somatic disease and reported that the association between depression and somatic comorbidity or geriatric syndromes was attenuated after adjustment for frailty. These findings were interpreted as suggesting that frailty may partially mediate or explain these associations. Both studies relied on cross-sectional data, and frailty was included as a covariate rather than formally modeled as a mediator, which limits causal interpretation. In contrast, our study conceptualized frailty as an effect modifier of the relationship between multimorbidity and depression, rather than as an explanatory or mediating factor. By stratifying analyses according to frailty status, we were able to identify a subgroup, frail individuals with multisystem physical multimorbidity, in whom depressive burden was markedly high. Specifically, frail participants within the multisystem multimorbidity class had six times the prevalence of depression compared with their apparently healthy counterparts. This approach aligns with the view of frailty as an integrative clinical phenotype reflecting cumulative vulnerability across physiological systems [50, 51]. Detecting frailty within multisystem multimorbidity classes may therefore be key to identifying individuals at particularly high risk of adverse health outcomes [52–54]. This subgroup may benefit from integrated, multi-component interventions tailored to address both physical frailty and mental health [55, 56].

Our study has several strengths. First, it used a data-driven latent class approach, allowing us to move beyond disease counts to identify patterns of multimorbidity. Second, the sample was large, probabilistic, and census-based, enabling us to conduct stratified analyses with frailty. Third, because exhaustion overlaps conceptually with depressive symptoms, our primary analyses used a modified four-criterion frailty phenotype excluding exhaustion. However, sensitivity analyses using the original five-criterion Fried phenotype yielded consistent results, supporting our findings. Despite strengths, our study has some limitations. We assessed a limited number of chronic conditions, omitting common conditions such a as cataracts, hearing loss, edentulism, or other important geriatric syndromes. Future research on multimorbidity should include a broader set of conditions to capture the full burden of aging-related health problems. Additionally, most chronic diseases were self-reported, which may lead to underestimation; however, evidence from previous studies supports the feasibility and reliability of self-reporting in epidemiological research [57]. An additional limitation relates to the requirement that participants be capable of performing spirometry. High-quality spirometry depends on adequate understanding, cooperation, and physical capability, which may be compromised in some older adults with advanced physical or cognitive impairment [58]. As a result, individuals with more severe limitations may be underrepresented in the sample, which should be considered when interpreting the generalizability of our findings. However, spirometry was conducted by trained and experienced fieldworkers, which may have reduced, but not eliminated, this potential source of selection bias. Moreover, we did not assess disease severity, which would have provided further characterization and depth to our multimorbidity classification. Finally, the cross-sectional design limits causal inference. Reverse causality is possible, as depression and multimorbidity are known to influence each other.

Our findings underscore the need to incorporate frailty assessments into the management of multimorbidity, particularly in mental health research and care. This aligns with the World Report on Aging and Health [8], which advocates shifting health systems from a disease-centered to a function-centered model. Recognizing frailty as a critical component of intrinsic capacity allows for more personalized, holistic care, especially in settings where healthcare resources are limited. To facilitate this shift, health information systems must be updated to include frailty and functional capacity indicators [59], enabling better identification, monitoring and management of at-risk older adults. Although our study offers cross-sectional insights, longitudinal research is needed to explore the temporal dynamics between multimorbidity, frailty, and depression. Reframing frailty as a central component in health care planning could help transform systems to more truly patient-centered environments [6].

## Conclusions

In this study of older adults from low-income urban areas in Lima, Peru, we identified three distinct patterns of physical multimorbidity: cardiometabolic, multisystem, and apparently healthy. Depressive symptoms were concentrated among individuals in the multisystem class, particularly among those who were frail. These findings highlight frailty as a key marker for identifying older adults with multisystem multimorbidity who are at elevated risk of depression. Our results support the need for function-focused, patient-centered care models in aging populations, especially in low- and middle-income countries. Incorporating frailty assessments into routine care may facilitate earlier identification and more targeted approaches to managing depression in later life.

## Supplementary Information


Supplementary Material 1.


## Data Availability

The GECo dataset used and/or analysed during the current study are available from the corresponding author on reasonable request.

## Abbreviations

AIC: Akaike Information Criterion
aPR: adjusted Prevalence Ratio
BLRT: Bootstrapped Likelihood Ratio Test
BMI: Body Mass Index
BIC: Bayesian Information Criterion
CES-D: Center for Epidemiologic Studies Depression Scale
CI: Confidence Interval
COPD: Chronic Obstructive Pulmonary Disease
FEV1: Forced Expiratory Volume in 1 Second
FVC: Forced Vital Capacity
GECo: Global Excellence in COPD Outcomes
LCA: Latent Class Analysis
LLN: Lower Limit of Normal
LMR: Lo-Mendel-Rubin Test
PASE: Physical Activity Scale for the Elderly
PHQ-2: Patient Health Questionnaire-2
PHQ-9: Patient Health Questionnaire-9
PR: Prevalence Ratio
SES: Socioeconomic Status
SD: Standard Deviation
