# Supplementary material for: Physical frailty, depression and multimorbidity among older adults in Lima, Peru: a cross-sectional study
Source: BMC Geriatr. 2026 Jun 20;26:1120. doi: 10.1186/s12877-026-07850-8 (PMC13536796; doi:10.1186/s12877-026-07850-8)
Supplement: Supplementary file 1 [file 12877_2026_7850_MOESM1_ESM.docx]

**Supplementary Material 1**

**Table S1**. Comparison of mean PHQ-9 scores between sequentially tested participants and those completing the Full PHQ-9

|  | **N (%)** | **PHQ-9 mean score** |
| --- | --- | --- |
|  |  |  |
| Full PHQ-9 | 993 (96.5) | 3.61 (4.45) |
| PHQ-2 | 36 (3.5) | 2.30 (3.76) |

*T-test (p<0.05)

**Table S2.** Prevalence ratio (PR) associations between physical multimorbidity classes and depression. Frailty definition includes 3 out of the 5 criteria (including exhaustion).

| **Class membership** | **Depressive symptoms (vs. No depressive symptoms)** | **Depressive symptoms (vs. No depressive symptoms)** |
| --- | --- | --- |
|  | Crude model | Adjusted model* |
|  | PR (95%CI) | PR (95%CI) |
| healthy | 1-reference | 1-reference |
| complex | **1.96 (1.11-3.46)** | **1.83 (1.04 – 3.22)** |
| metabolic | **1.43 (1.05-1.94)** | **1.35 (1.00 – 1.84)** |
| **Among those with frailty** |  |  |
| healthy | 1-reference | 1-reference |
| multisystem | **3.16 (1.63-6.16)** | **3.36 (1.34-8.46)** |
| metabolic | 1.18 (0.57-2.48) | 1.43 (0.73-2.78) |
| **Among those without frailty** |  |  |
| healthy | 1-reference | 1-reference |
| multisystem | 1.68 (0.85-3.30) | 1.52 (0.79-3.00) |
| metabolic | **1.42 (1.02-1.99)** | 1.32 (0.95-1.85) |

Model adjusted by sex, age, educational level and socio-economic position.
